# Origins of the arctic fox variant rabies viruses responsible for recent cases of the disease in southern Ontario

**DOI:** 10.1371/journal.pntd.0007699

**Published:** 2019-09-06

**Authors:** Susan A. Nadin-Davis, Christine Fehlner-Gardiner

**Affiliations:** National Reference Laboratory for Rabies, Ottawa Laboratory–Fallowfield, Canadian Food Inspection Agency, Ottawa, Ontario; US Department of Agriculture, UNITED STATES

## Abstract

A subpopulation of the arctic fox lineage of rabies virus has circulated extensively in red fox populations of Ontario, Canada, between the 1960s and 1990s. An intensive wildlife rabies control program, in which field operations were initiated in 1989, resulted in elimination of the disease in eastern Ontario. However in southwestern Ontario, as numbers of rabid foxes declined the proportion of skunks confirmed to be infected with this rabies virus variant increased and concerted control efforts targeting this species were employed to eliminate the disease. Since 2012 no cases due to this viral variant were reported in southwestern Ontario until 2015 when a single case of rabies due to the arctic fox variant was reported in a bovine. Several additional cases have been documented subsequently. Since routine antigenic typing cannot discriminate between the variants which previously circulated in Ontario and those from northern Canada it was unknown whether these recent cases were the result of a new introduction of this variant or a continuation of the previous enzootic. To explore the origins of this new outbreak whole genome sequences of a collection of 128 rabies viruses recovered from Ontario between the 1990s to the present were compared with those representative of variants circulating in the Canadian north. Phylogenetic analysis shows that the variant responsible for current cases in southwestern Ontario has evolved from those variants known to circulate in Ontario previously and is not due to a new introduction from northern regions. Thus despite ongoing passive surveillance the persistence of wildlife rabies went undetected in the study area for almost three years. The apparent adaptation of this rabies virus variant to the skunk host provided the opportunity to explore coding changes in the viral genome which might be associated with this host shift. Several such changes were identified including a subset for which the operation of positive selection was supported. The location of a small number of these amino acid substitutions in or close to protein motifs of functional importance suggests that some of them may have played a role in this host shift.

## Author summary

Rabies, a serious disease which almost invariably results in death once clinical signs appear, is caused by the rabies virus. The arctic fox lineage of rabies virus persists in fox populations in northern Canada and from the 1960s onwards a sub-population of this virus has circulated in red foxes in the province of Ontario, Canada. By 2000 a provincial wildlife control program attempting to control fox rabies in southern Ontario had eliminated the disease in most of the targeted regions except for a focus of rabies in certain counties in southwestern Ontario which involved mostly skunks. Modified control efforts resulted in apparent rabies elimination with no cases reported in southern Ontario after May 2012 until a rabid bovine was identified in December 2015. Additional cases, mostly in skunks and livestock, have been reported subsequently. To discriminate between a re-introduction of the virus from the north and re-emergence of the Ontario viral variant, we sequenced and compared the genome of multiple viruses from both areas. The sequence data indicate that sub-types of the Ontario variant which eluded control efforts are responsible for these recent cases. These data also permit an examination of potential genetic changes in the virus that may facilitate its circulation in this new wildlife host, the skunk.

## Introduction

Rabies virus (RABV), the type species of the *Lyssavirus* genus, is the most commonly encountered etiological agent of rabies, a serious zoonosis considered virtually 100% lethal once clinical signs are apparent [1]. This neurotropic agent propagates in the central nervous system of its victim and causes significant behavioural changes, encephalopathy and eventual death [2]. The relatively small viral genome of 12 Kb encodes just five proteins which have a highly modular structure due to their multi-functional nature [3]. The nucleoprotein (N) encapsidates the RNA genome and is an essential component for viral transcription and propagation together with the Large protein or polymerase (L), which provides all enzymatic functions, and the phosphoprotein (P) cofactor. The P protein is also an important regulator of the host immune response. The matrix protein (M) has both regulatory and structural functions while the glycoprotein (G), the sole surface protein of the viral particle, is responsible for host receptor binding and viral entry into the cell as well as being the primary factor to elicit the generation of protective antibodies [3]. Similar to other RNA viruses which employ a polymerase which lacks proofreading activity [4], RABV exhibits high mutation rates resulting in continuous evolution of the virus such that once it is established in a new reservoir host it quickly evolves into a variant that is genetically distinct from other rabies virus populations [5]. Accordingly distinct viral variants, each associated with a particular host species that can efficiently spread the disease, are distributed across much of the globe [6]. In Canada, wildlife species, including foxes and skunks, remain the principal reservoir hosts for rabies. Across northern Canada arctic foxes maintain the arctic fox rabies virus lineage (AFX RABV), transmission of which into red fox populations has allowed its spread into southern Canada on multiple occasions. In the 1950s and 1960s an epizootic of AFX RABV spread into southern and eastern Ontario where it became permanently established in red fox populations [7]. Throughout the 1970s and 1980s rabies cases reported across the province averaged 1500–2000 annually; the enzootic was maintained primarily by red foxes but the disease spilled over into many other wildlife species, most notably skunks, as well as domestic animals [8].

**Fig 1:**
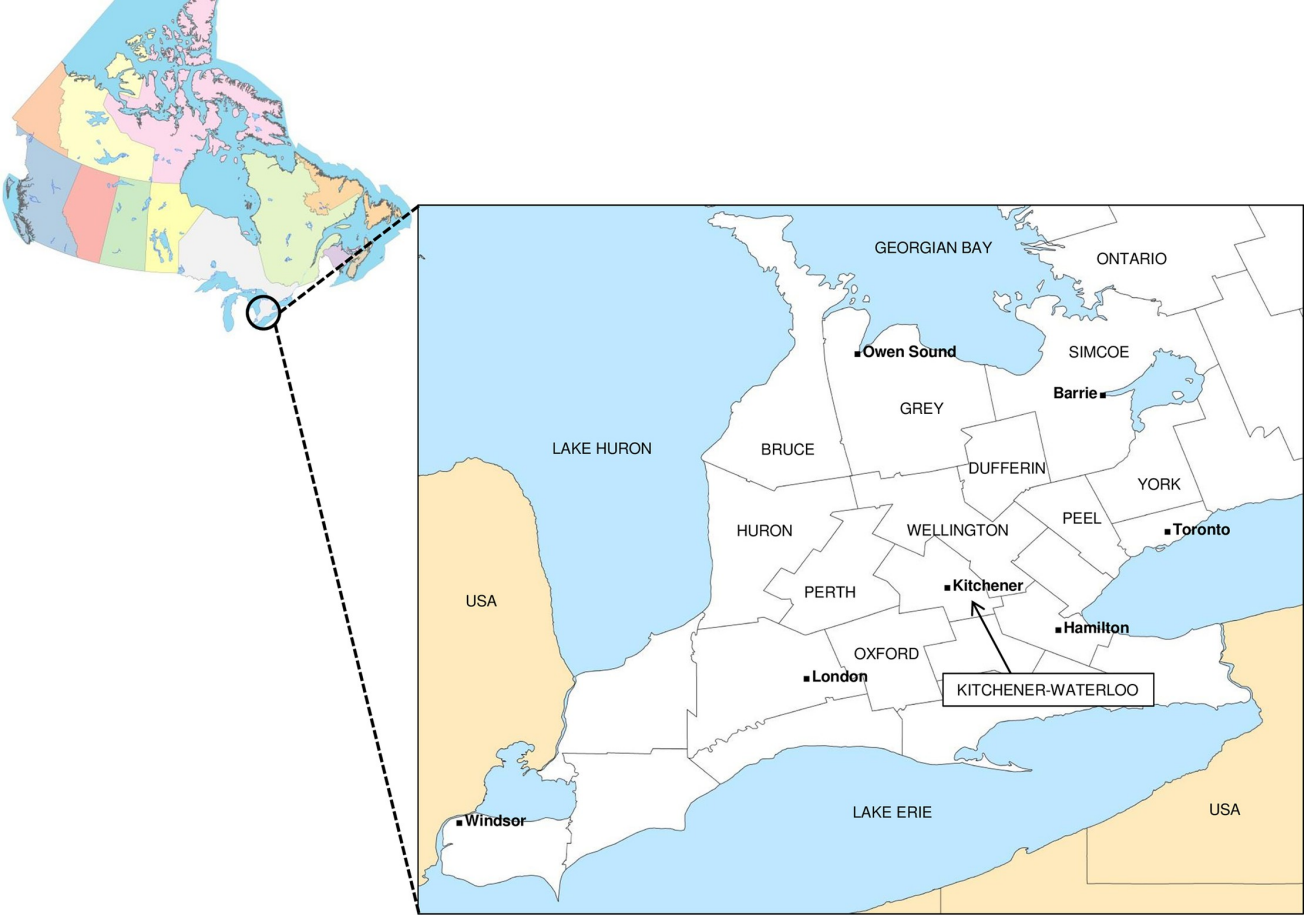
A map of Canada and insert showing the location of the southwestern Ontario study area. Grey lines define county boundaries and the 11 counties included in the study are identified. Cities and major bodies of water are also labelled.

Due to the public health and animal welfare concerns of continued circulation of rabies in wildlife, the province of Ontario developed an intensive control program based upon oral rabies vaccination (ORV) which aimed to eliminate the disease from the fox populations of eastern and southern Ontario through annual aerial distribution of vaccine-laden baits. Fox rabies was eliminated from eastern Ontario in the 1990s [9] but this control program was met with additional challenges in several counties of southwestern Ontario (Fig 1). Despite annual ORV campaigns in parts of this region since 1994 which appeared to succeed in reducing fox rabies, a disease focus driven primarily by the striped skunk persisted. Modification of rabies control efforts, including the development and deployment of a new ONRAB vaccine, based on a recombinant adenovirus construct, to better target skunks, did appear to finally successfully eliminate AFX RABV from the area [10,11]; in 2012 only two cases were reported in southwestern Ontario (one striped skunk and one domestic animal) and none was reported in 2013 and 2014. With no cases reported in southwestern Ontario in terrestrial species for two years, in June 2014 the area met the WHO definition of freedom from terrestrial rabies and accordingly the annual ORV program was discontinued in this region.

However, in December 2015, after a gap of 3.5 years, a single rabies case due to the AFX RABV variant virus was identified in a bovine from Perth County. In 2016 a second bovine case in the same county was recorded together with two cases in skunks. Four additional bovine cases occurred in 2017 together with six wildlife cases (five skunks and one red fox) and another seven cases (six skunks and one bovine) were identified in 2018 (https://www.ontario.ca/page/rabies-wildlife). The affected area has expanded into Huron, Kitchener-Waterloo and Wellington-Dufferin-Guelph counties and many of the wildlife cases were initially identified due to enhanced surveillance by provincial authorities. All cases were typed by antigenic methods as belonging to the AFX RABV variant; however this method does not discriminate between those viruses that circulate in northern Canada and those previously identified in southern Ontario.

Previous genetic analysis of the AFX RABV lineage identified four main sub-lineages, one of which (A1) is represented by viruses recovered in southern Ontario only while another (A3) identifies all the viruses now circulating in northern Canada [12]. Early studies of the A1 sub-lineage targeting individual genes identified four main variants (ON1-4) circulating in localised areas of Ontario [13]. ON1 was found exclusively in eastern Ontario while variants ON2-4 circulated in overlapping ranges within southwestern Ontario. Since those studies, the development of highly parallel methods of sequencing now facilitate efficient generation of whole viral genome sequences [14] which allow discrimination between very similar viral variants and can reveal the evolution of the virus as it spreads across the landscape [15,16]. Such an approach was ideally suited to the analysis of the current situation in southern Ontario. Accordingly this study has explored the detailed phylogenetic structure of the AFX RABV circulating in 11 counties of southwestern Ontario since the 1990s through to the present with comparison to representative samples from eastern Ontario and the Canadian north. This analysis shows that the present outbreak of AFX RABV in southwestern Ontario is a continuation of the enzootic that has persisted in the province since the 1960s and is not due to a new incursion. Challenges in surveillance that occurred during the recent study period may have contributed to an apparent gap in identification of cases. The detailed genetic analysis of these viruses has also permitted an examination of changes in the virus which may have occurred as a result of the apparent host shift from red foxes to skunks. Interrogation of the genome sequences generated during this study identified several coding changes that occurred during the evolution of the AFX RABV variants in Ontario. Some of these changes were identified as having undergone episodic positive selection and their potential role in host adaption is discussed.

## Materials and methods

### Rabies virus samples

All brain tissue samples included in this study (see S1 Table) had been previously diagnosed as rabies positive by the direct fluorescent antibody test (FAT) applied to brain smears [17] by the CFIA’s National Reference Laboratory for Rabies located in Ottawa, Ontario. In some instances the samples had originally been collected as part of the Ontario Government’s enhanced surveillance activities in support of rabies control operations and diagnosed as positive using the Direct Rapid Immunohistochemical Test (DRIT) for rabies [18]; these samples were subsequently submitted to the CFIA for confirmatory testing using the FAT. All positive cases were subjected to viral antigenic typing performed as described [19] and brain material from these cases was maintained in long-term storage at -80°C.

### RNA extraction and viral genome amplification

RNA extracted from brain tissue during previous studies had been prepared using TRIzol according to the supplier and held in long term storage at -80°C. Samples newly extracted for these studies were prepared using a hybrid method in which the aqueous phase from the TRIzol extraction was further purified using an AMBION 1830 RNA extraction kit with a MagMax 96 deep well system as described [14]. Extracts were quantified spectrophotometrically using a Nanovue system. Amplification of whole viral genomes was accomplished by generation of overlapping RT-PCR products generated by a modified version of a previous method [14]. Redesigned primers to support efficient amplification of the AFX RABV were used for both first round PCR, to generate three overlapping products (A, B and C), and a second round of hemi-nested PCR (hnPCR) as needed (S2 Table). Reverse transcription was performed on 2 μg RNA using random primers and 5 μl aliquots of the resulting cDNA were used in a 50 μl first–round PCR containing 1x HiFi buffer with Mg++ (supplied with the enzyme), 0.2 mM dNTPs, 0.3μM of each primer and 1 unit of Phusion HiFi Hot start DNA Polymerase (ThermoFisher). Thermocycling conditions for amplicons A and B were 95°C for 4 min followed by 40 cycles of: 98°C, 20 s; 65°C, 15 s; 72°C, 5 min and a 72°C hold for 5 min. The thermocycling profile for amplicon C was similar except that the extension temperature was reduced to 48°C for the first two cycles and then increased only to 55°C for the remaining 38 cycles. If required hnPCR was performed using 2 μl of first round product and similar conditions except that PCR was performed for 25 cycles with an annealing temperature of 55°C. Agarose gel electrophoresis was used to verify production of amplicons which were then purified using a Genejet PCR purification system (ThermoFisher) prior to sequencing.

### Sequencing and phylogenetic analysis

Whole genome sequencing (WGS) of all RABV samples was achieved using Illumina technology as previously described [14]. Amplicons from each individual sample were quantified, pooled and processed in batches of 96 using a Nextera XT DNA library kit and normalised libraries were sequenced on a MiSeq instrument using either 2x250 or 2x300 run kits. Resulting fastq files were subjected to a reference based assembly using the DNASTAR v14 software package and an AFX RABV whole genome sequence determined previously (NCBI Accession # KU198468) as reference; final consensus sequences were exported in fasta format.

All fasta files were aligned using the MUSCLE algorithm implemented in MEGA 7 [20] to generate an alignment of 11935 bases. The GTR+G nucleotide substitution model was found to best fit this alignment using Modeltest and the data were accordingly used to generate a Maximum Likelihood (ML) tree with MEGA 7 in which clades identifying distinct variants were defined using a genetic distance >0.005. A time-scaled phylogeny of the WGS data was constructed using the BEAST v1.7.5 package [21] with the BEAGLE algorithm [22] and employing the GTR+G model of nucleotide substitution with a relaxed molecular clock model. Two independent runs, each of 50 million MCMC iterations with 10% burn-in, were performed and convergence of the results verified using Tracer v 1.6 software. Results were summarised as a maximum clade credibility (MCC) tree using TreeAnnotator v1.7.5, and visualised using Figtree v1.4 (available at http://tree.bio.ed.ac.uk/software/figtree/).

### Exploration of changes associated with adaptation to the skunk host

Using MEGA v7 the complete WGS dataset was edited to remove all non-coding sequence and thereby create an alignment of all five concatenated ORFs using an A3 sub-lineage isolate (NT.1991.0085AFX) as the reference. To explore any evidence for positive selection, this alignment was analysed using a mixed effects model of evolution (MEME) [23], capable of identifying episodic selection operating on individual sites, implemented through the on-line analysis tool “datamonkey” (available at www.datamonkey.org/) using a cutoff of P<0.1. The analysis employed a ML tree generated from the edited file that yielded a phylogeny similar to that produced using the complete WGS data. Based on this alignment all amino acid substitutions compared to the reference that were present in three or more samples were catalogued and those considered of special significance due to operation of selection or location within significant protein motifs were selected for further comparison with corresponding residues of a skunk-adapted sample (A10-0514, NCBI accession # JQ685938) of the south central skunk variant (SCSK).

### Geographic mapping

The town or city from which each sample originated was used to determine latitude and longitude co-ordinates for mapping. Data points of RABV subtypes were mapped using the ARC-GIS v10 software.

## Results/Discussion

### Arctic fox variant rabies cases in southwestern Ontario

**Fig 2:**
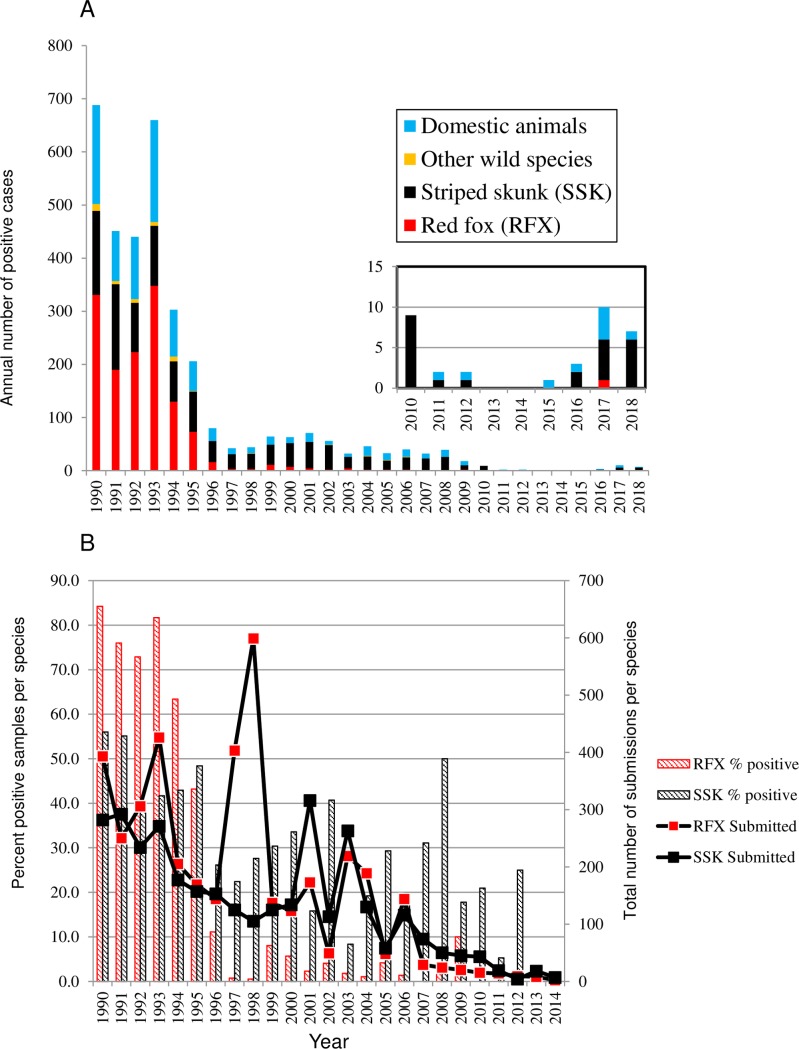
Panel A. Summary of rabies cases due to the AFX variant in 11 counties of southwestern Ontario 1990 to May 2018. The inset shows the recent case data from 2010 onwards at a different scale. Panel B. Total submissions and the percentage of all submissions testing rabies positive by year for the red fox (RFX) and skunk (SSK) hosts within the study zone. Data beyond 2014 are not shown as submissions in recent years were extremely low and sporadic.

The area of southwestern Ontario which was the focus of this study is comprised of 11 counties located in the most southern part of the province bound to the south and west respectively by the Great Lakes Erie and Huron (Fig 1). Rabies case reports caused by all AFX RABV variants recovered in this study area from 1990 to the present are summarised in Fig 2. The significant overall decline in rabies cases between 1990 and 1996 (Fig 2A) reflects the success of the provincial ORV program targeting foxes [9,24] and the concomitant effect of increasing immunity within the fox population on reduced spill-over to other species. This reduction in rabies cases was mirrored by an overall decline in the annual number of total submissions for this area, which dropped from over 2000 in the early 1990s down to a few hundred by the year 2010. Moreover this drop was accompanied by reductions in the percentage of submissions testing positive for both foxes and skunks (Fig 2B). However as total case numbers fell, the proportion of cases in skunks rose from a mean of 24.4% in the years 1990–1994 to 71.4% by the year 2000 and from 2002 onwards skunks made up the vast majority of wildlife cases. Also notable was the significant number of infected bovines which frequently constituted the largest group of domestic animals that were reported.

The apparent shift in reservoir species for the AFX RABV in southwestern Ontario had prompted modification of rabies control efforts including deployment of a new vaccine, ONRAB, to target skunks [11]. This approach appeared to have the desired effect with zero cases of rabies due to AFX RABV reported in southwestern Ontario in 2013 and 2014. As a result from 2014 onwards submissions were highly sporadic as the disease was believed to have been eliminated. However since 2015 additional cases due to this viral variant have been identified (Fig 2 inset). Notably just one bovine case was reported in 2015 with further cases in both bovines and skunks the following years as well as one positive result in a red fox.

Since the antigenic method routinely used to type these viruses is unable to discriminate between distinct sub-lineages and variants of AFX RABV it was unclear whether these recent cases were the result of re-emergence of an Ontario variant of the A1 sub-lineage or the result of a new incursion, perhaps due to a translocation of a diseased animal from a northern community carrying the A3 sub-lineage. Genetic analysis of the rabies virus to better understand the origins of these cases was therefore undertaken.

### Viral phylogeny

**Fig 3:**
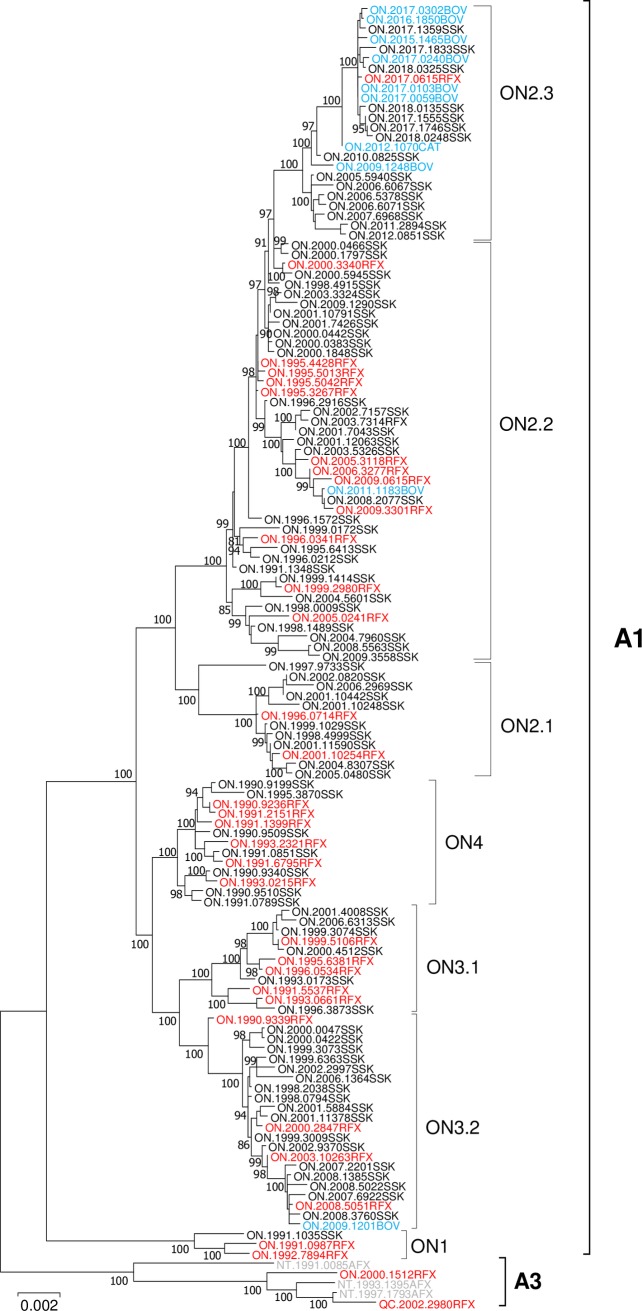
A ML tree generated using WGS for 133 AFX RABVs. The tree was generated using the GTR+G nucleotide substitution model best supported by the data with 200 bootstrap replicates. Bootstrap values for all major nodes having values ≥ 80% are indicated and a distance scale is shown below the tree. Groups and clades as described in the text are identified to the right of the tree. Sample names are color-coded according to the host species thus: red fox, red; skunk, black; domestic animals, blue; arctic fox, grey.

**Fig 4:**
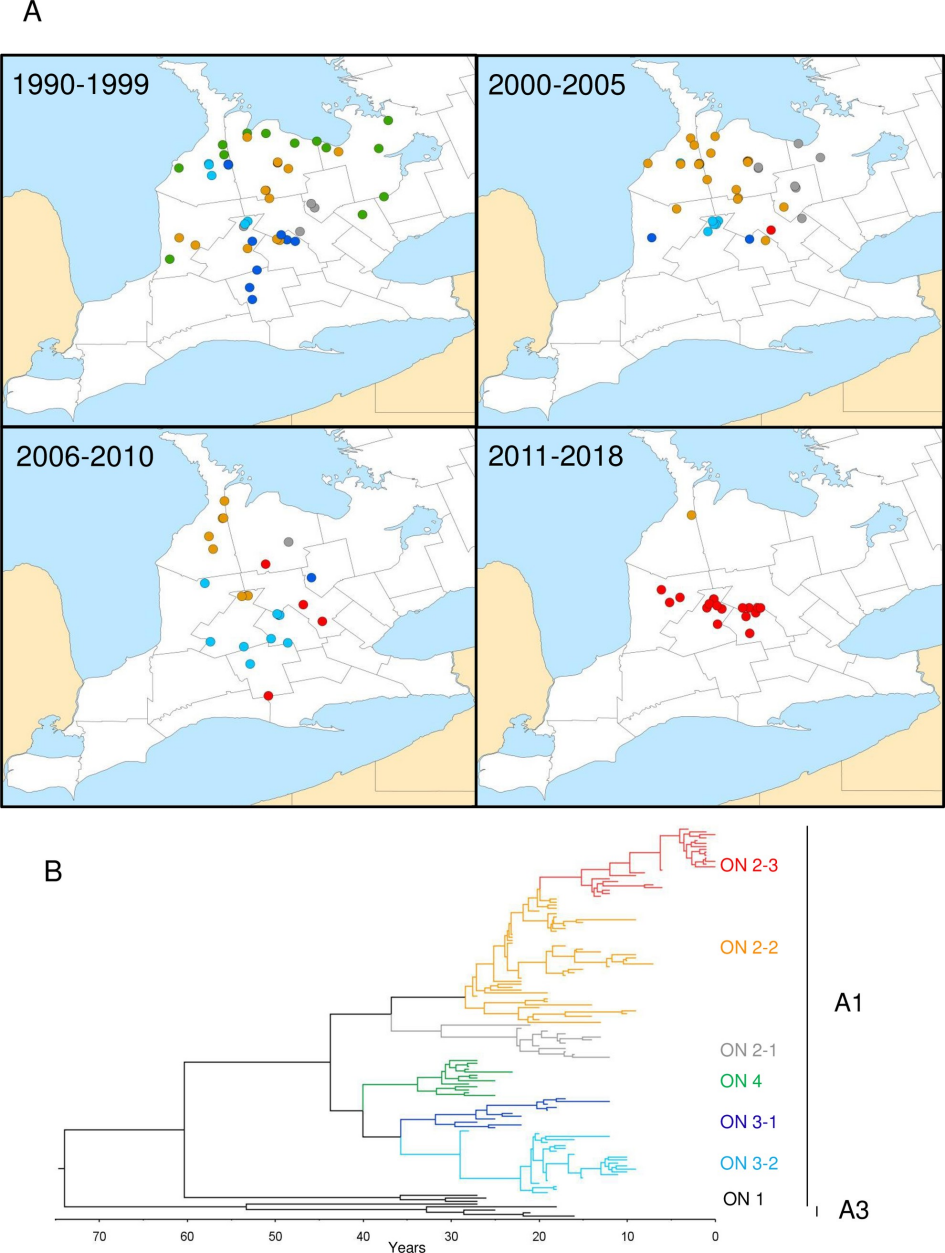
Maps of the study area showing the locations of all RABV variants over four time periods (panel A). Maps were generated using ARC-GIS software, v. 10. RABV variants and sub-variants are identified by colour-coding as illustrated in the time-scaled MCC tree generated using the BEAST software package (panel B).

WGS data were generated for 128 samples, collected since the 1990s from southern and eastern Ontario and confirmed to be infected with AFX RABV by antigenic analysis. A phylogenetic tree constructed from these data together with five representatives of the A3 sub-lineage from northern Canada (Fig 3) clearly differentiates between the northern A3 and Ontario A1 sub-lineages. Based on their genetic distances (>0.005) the Ontario samples were further divided into four major clades representing variants ON1 to ON4 consistent with previous findings [13]. The ON1 variant, which diverged from all other Ontario AFX RABV variants soon after its introduction into the region, is comprised of three samples, previously classified by partial sequencing within this group, which were recovered from eastern Ontario only. All samples of the remaining three clades, representing variants ON2 to ON4, were recovered from within the southwestern Ontario study area. The ON2 and ON3 variants were further subdivided into distinct sub-variants identified by well-defined and well supported clades as shown (Fig 3). The geographical distribution of all viral types over four time periods within the study area is illustrated together with a time-scaled tree generated using this same dataset (Fig 4). Although the pairwise genetic distances between the 125 samples from the study area was relatively small, ranging from 0.000 for two 2017 bovine samples to 0.0184 for two skunk samples collected nine years apart, such differences are considered robust. Despite the large number of amplification cycles performed during sample processing and sequencing, prior evaluation of this method through replicate independent sample analysis has shown that it yields highly consistent consensus sequences [14].

Based on the time-scaled phylogeny, ON3 and ON4 variant viruses emerged from a common progenitor about 40 years ago (circa 1978). The thirteen ON4 viruses form a relatively homogeneous group recovered primarily along lakeshore areas south and east of Georgian Bay in Grey and Simcoe counties, but also from as far south and west as communities in York, Peel, Bruce and Huron counties. The samples recovered close to Lake Huron were previously classified by restriction fragment length polymorphism (RFLP) only and exhibited patterns between variants ON2 and ON4 [13]; this more thorough analysis clearly demonstrates their association with the ON4 clade thereby identifying a broader circulation of this variant than had been recognised previously. However, samples of this variant were identified in the 1990s only; indeed, the last ON4 isolate was recovered in 1995 and it would appear that this viral variant has not circulated in the area for some time.

The ON3 variant consists of two sub-variants, ON3-1 and ON3-2, which diverged about 35 years ago (1983). ON3-1 samples were recovered from counties central to southwestern Ontario (mostly from Oxford and Kitchener-Waterloo) with spread into many neighboring counties (Perth, Huron, Dufferin and Bruce). Most isolates of this type were recovered prior to 2000 with the last identified in 2006. A greater number of ON3-2 samples were recovered, mostly from Perth and Bruce counties up until the early 2000s with later southwards spread into Kitchener-Waterloo and Huron counties where the last isolates were recovered in 2008 and 2009.

As evident by the phylogeny of this group, the ON2 variant which diverged from the other variants about 44 years ago (1974) is the most heterogeneous and persistent, continuously evolving throughout the study area. Members of ON2 fall into three distinct clades: a smaller group (ON2-1) was found in Wellington, Dufferin, Grey and Simcoe counties between 1996 up until 2006, spreading into the northeastern part of the study area where ON4 had circulated previously. The large heterogeneous ON2-2 sub-variant which circulated extensively throughout the study area eventually disappeared with the last case identified in Bruce County in 2011. However, the ON2-3 sub-variant that first emerged about 15 years ago (2003) was initially distributed primarily within Wellington and Kitchener-Waterloo counties but has subsequently been recovered more widely and is the only viral type recovered in southwestern Ontario since 2012. The finding that all samples from 2015 onwards recovered from southwestern Ontario carry a virus that clusters within the ON2-3 clade of the ON2 variant clearly demonstrates that this new outbreak is the result of re-emergence of the viruses that circulated in the study area previously and rules out any re-introduction of rabies from elsewhere.

These results clearly demonstrate that, while the provincial control program effectively eliminated both the ON3 and ON4 variants circulating in southwestern Ontario, the ON2 variant persisted in some areas and has evolved into the viral sub-variant (ON2-3) now being recovered exclusively. Programmatic changes in 2014, which shifted rabies suspect sample collection and submission activities from federal to provincial jurisdiction, may have contributed to a gap in surveillance which allowed continued presence of the virus to go undetected for 3.5 years. During the transition of the program in 2014 and 2015, rabies sample submissions to the CFIA Ottawa Laboratory-Fallowfield from the southwestern Ontario area were 400–500 samples per year, down from 1000–1200 per year in the three years prior to the program change. In 2016, submission numbers returned to previous levels (~1100 samples tested). It is notable however that apart from the bovine cases reported from 2015 onwards, all the skunk cases were identified as a result of the province of Ontario’s enhanced surveillance efforts initiated in response to the bovine cases as well as a large outbreak of raccoon rabies that has spread from its origins in the city of Hamilton, Ontario, located to the southwest of the study area. Only one wildlife case, a red fox, was identified in these last two years as a result of the routine passive surveillance efforts normally employed in the province.

### Viral adaptation to the skunk host

Given that the vast majority of cases of rabies in terrestrial wildlife species in southwestern Ontario over the last 20 years have involved the skunk it would appear that the AFX RABV variant has effectively undergone a host switch from the red fox to this new host. Indeed a review of the host distribution throughout the viral phylogeny (Fig 3) illustrates the high proportion of skunks compared to foxes within the ON2 variant especially for samples collected in the 21^st^ century. A similar trend appeared to be emerging for the ON3-2 sub-variant until its elimination. Such a host switch may indeed have been driven by the control efforts that initially targeted foxes to generate an immune fox population combined with significant fox-skunk interactions of these sympatric species in the area and the skunk’s relatively high capacity to maintain several other RABV variants across regions of North America [25]. While such “host jumps” by rabies viruses are considered quite rare they have been documented on occasion and form an important basis for the evolution of the virus [5,26,27] but the mechanism underlining these events remain unclear. A few studies have found evidence in support of positive selection on diverse sites in genes encoding the glycoprotein, nucleoprotein and polymerase products during RABV adaptation to new host species [28,29] while other studies have failed to identify the operation of positive selection during host shift events [26]. The wealth of sequence data generated in this study over a long period of time did however offer a unique opportunity to re-examine this process in detail.

Due to the restricted range of the skunk to southern Canada it was assumed that the viruses of the A3 sub-lineage, which circulate in foxes in the north, would not exhibit any skunk-adapted traits. Accordingly, alignments of the entire 133 sample database using an A3 isolate (NT.1991.0085) as reference were generated and used to predict amino acid coding differences between the Ontario A1 variants and the northern A3 sub-lineage viruses. Using an alignment of concatenated sequence for all five ORFs, evidence for the operation of positive selection on individual sites was explored using MEME, which is specifically designed to test for episodic and pervasive selection at individual sites even when such selection is not applied to all taxa [23]. MEME analysis identified 161 sites within the alignment that were under episodic selection (P<0.1) (S3 Table). While many of these sites exhibited substitutions in just single isolates others were represented by larger groups of samples and all amino acid differences from the reference that occurred in more than two isolates from any species are identified in S4 Table. Table 1 summarises the most notable amino acid substitutions identified by one or both of these approaches given either the position within the phylogeny at which the change occurred or the location of the change within or close to protein motifs of known or suspected function. A comparison of these changes with the amino acid present in the corresponding position for an isolate of a phylogenetically distinct skunk-associated RABV, the south central skunk (SCSK), is also included.

**Table 1:** AFX RABV ORFs and notable amino acid substitutions in Ontario RABV samples compared to a virus of the A3 sub-lineage.

ORF	Length (residues)	Residue position	Amino acid Substitution^1^	Functional domain	Samples in which change observed	Residue in SCSK variant^2^
N	450	332*	A to T	Upstream of antigenic site I	Three isolates of ON3-2	T
		379*	T to A or V	Contained within site IV epitope; close to Ser_389_ phosphorylation which has important regulatory effects	A in most ON samples; V in one ON2-2 sample (ON.2005.0241RFX) and all ON2-3 (except M in ON.2007.6968)	V
P	297	48	N to H	Immediately adjacent to a nuclear export signal at sites 49–58	In later ON2-2 isolates and all ON2-3	S
		85*	E to G	Located in a disordered less conserved domain	Present in all ON2-3	E
		160*	I to V or A	Immediately upstream of Ser_162_ that acts as a phosphorylation site	I in all A3 and ON1; V in all others except A in 3 members of ON2-2	S
		174*	V to A	Just downstream of the dynein binding motif	In 5 samples: one A3, all ON1 and ON.2012.0851 (L in ON.1990.9509)	A
		295*	T to A	Terminal region potentially involved in N protein binding	In all A1 sub-lineage	L
		297	S to C	Terminal residue potentially part of the N protein binding motif	In ON1 only	N
M	202	51	K to R	Unknown	In all A1 sub-lineage	R
G—mature product	505	29*	V to I or M	Downstream of the N-terminal linear epitope (14–19)	I in two later isolates of ON2-2, I or M in ON2-3	V
		193	T to I	Upstream of AS II (198–200)	In 17 later isolates of ON2-3 only	T
		255	D to N	Upstream of AS IV (263–264)	In later members of ON2-2 and all ON2-3	D
L	2127	207*	I to V	Upstream of block I	In later ON2-2 and all ON2-3 (T in ON.1993.0173)	M
		350	G to R	In block I	In all ON2	K
		883*	S to N	Upstream of block IV	In all ON2-2 and 2–3	N
		1140*	S to N	In block V	In all ON2	G
		1264*	I to V	In block V	In all ON2 except ON2-1 and early ON2-2	V
		1276	Q to H	In block V	In ON1 only	H
		1277	D to E	In block V	In all ON2-3 samples from 2015 onwards	D
		1426*	V to I	In interblock V-VI	In two members of ON1 (both fox isolates)	V
		1564	A to D	In interblock V-VI	In all except A3 sub-lineage and ON1	V
		1615	T to A	In interblock V-VI	In ON1 only	A
		1623	N to H	In interblock V-VI	In all ON2-3 samples from 2012 onwards	N
		1950*	K to R	In C-terminal region	In all except A3 sub-lineage and ON1	K
		2046	N to T	In C-terminal region	In later ON2-3 only	N
		2089	I to V or S	In C-terminal region	S only in all ON2-3 samples from 2015 onwards; V in four ON4	V

^1^ Compared with reference sequence NT.1991.0085 (A3 sub-lineage)

^2^ Based on sequence of south central skunk variant isolate A10-0514 (NCBI accession # JQ685938)

*Residues identified as undergoing episodic selection

Many of the amino acid substitutions listed in Table 1 are highly conservative and involve residue changes with similar properties such as changes involving amino acids with nonpolar side groups. While these could simply be neutral mutations that maintain the protein’s required 3D structure, the residue’s altered size could have functional significance especially if the substitution occurs close to motifs of known function. For instance, the substitutions in the phosphoprotein found in most Ontario isolates except ON1 involve an Iso_160_ to Val_160_ change immediately upstream of Ser_162_, an important phosphorylation site; residue 160 was a serine in the SCSK sample. A Val_174_ to Ala_174_ change located just downstream of the dynein binding motif [30] was notable although it occurred in just five samples and appears unrelated to the emergence of ON2. Other mutations involving changes to the nature of the amino acid side chain may have an even greater impact on protein structure and function, particularly if they are located in or around motifs of known function. The substitutions observed at position 379 in the nucleoprotein occur within an antigenic site and are close to Ser_389_, a phosphorylation site with regulatory effects on virus replication [3,31]. Notably the SCSK sample contains a valine in this position similar to that observed for many of the later ON2 samples. While residue 48 of the P protein was not identified as being under selection by MEME (P = 0.37), an Asn to His substitution is present in many of the later ON2 isolates; this residue is immediately adjacent to a nuclear export signal at sites 49–58 [30]; this site together with a nuclear localisation signal regulates trafficking of the P protein isoforms into the nucleus where they can interfere with the host interferon response [32]. A serine occupies this site in the SCSK sample. Changes at residues 29, 193 and 255 of the glycoprotein, located in the vicinity of several antigenic sites [33] [34] also involve certain later ON2 variants but the latter two were not identified as being under selection and given that the SCSK samples retained the original amino acid at all of these positions these substitutions may thus represent neutral mutational drift. A number of coding changes within the L gene encoding the polymerase responsible for RNA synthesis, capping and other enzymatic functions are also noteworthy. This protein has a modular structure consisting of six well- conserved regions (blocks I to VI) responsible for its enzymatic functions separated by less conserved regions [35]. Variability within the current data set was especially notable within blocks I and V and the more variable region separating blocks V and VI with a number of less conservative substitutions noted in these areas that distinguish all southwestern Ontario samples and especially those of the ON2 variant. Indeed it has been suggested previously that the variable nature of the inter-block V-VI region may reflect specialised functions of this region [35] and thus potentially involve interaction with host factors. The observation that some of these substitutions have emerged within the ON2 variant under positive selection renders these changes as good potential candidates for contribution towards host adaptation. In particular the amino acids of many members of the ON 2 variant at L protein positions 350, 883, 1264 and 1426 match those for the SCSK sample. Interestingly other changes closer to the C-terminus of L do not fit with a pattern in which the skunk-associated AFX and SCSK variants are converging. It remains likely of course that host adaptation may involve several sites evolving over time that collectively provide the virus with greater fitness to persist in a new environment.

The use of the MEME method, which has been reported to be far better at identifying sites under positive selection compared to other methods such as FEL [23,36], was critical to successful identification of such sites within the database under study. Analysis of this same database using FEL failed to identify any sites under positive selection. It remains possible that some of the sites identified here are false positives given use of the P<0.1 cut-off although most the sites reported here were supported at the P<0.05 level. Failure to identify positive selection in other studies [26,37] may thus be a result of the methods employed rather than the absence of selective forces.

## Conclusions

The experience of the Ontario wildlife rabies control program described here underlines the importance of ongoing comprehensive surveillance in areas undergoing rabies control. Although programmatic changes did reduce the number of submissions for rabies diagnosis in 2014 and 2015 a degree of surveillance was maintained and yet failed to identify any cases. The apparent elimination of AFX rabies in the area over a two-year period led to the self-declaration of a terrestrial rabies-free status for southwestern Ontario and discontinuation of control efforts. In retrospect the claim of rabies freedom for the area was premature as this study has established that the virus responsible for the most recent cases had evolved from previously circulating variants. Indeed, as an area approaches successful rabies elimination establishing beyond reasonable doubt that the enzootic has been eliminated becomes challenging due to the very low numbers of cases likely to be encountered. In this situation a two year period without any cases proved to be insufficient to establish complete eradication of the disease. It is unknown if continuation of the ORV program for one or two more seasons would have been more successful but authorities involved in rabies control, especially in wildlife given its limited human interaction, may consider continuing with vaccination activities beyond a two year window during which no cases are identified. The alternative may be a more aggressive active surveillance program to help reveal small pockets of disease persistence. In this respect the bovine, which is not a rabies reservoir host, appears to be a useful sentinel species given that re-emergence of AFX RABV was first detected in these animals. Compared to companion animals, for which vaccination is mandatory in Ontario, cattle are often unvaccinated and have a higher chance of being exposed to the disease through contact with wildlife when in pasture. Moreover, the high value of many bovines and the compensation packages available to farmers for animals diagnosed as rabid provide incentives for their submission for rabies testing.

The other notable observation in this study is the host shift event that has confounded control efforts given that modifications to the baiting program were required in order to target the skunk host compared to the red fox. Furthermore, the identification of one case in a red fox in southwestern Ontario in 2017 raises the spectre of more widespread reintroduction of the disease into this highly susceptible host from the skunk reservoir. While host shifts are not considered common, there is mounting evidence that it has occurred on many occasions over time, in particular from dogs to wildlife species [37]. Such situations can pose major challenges to control efforts, especially in those regions where dog rabies is endemic since other host species not targeted in a campaign may harbor virus capable of being re-introduced into the dog population. Surveillance to gather knowledge of the circulation and persistence of rabies in potential host reservoirs in any control zone will be valuable to an informed control program.

What enables RABV to adapt to new host reservoirs remains poorly understood possibly because the process is multifaceted, involving changes at a number of coding positions, and thus difficult to identify using current approaches. Analysis of the 3D structure of viral proteins, posttranslational modifications, and better knowledge of the host factors with which these products interact may help us better understand the association of rabies virus variants with specific host species. The information provided in this report may help to direct such future studies to particular protein motifs of potential importance in this regard.

## Supporting information

S1 Table List of all rabies virus samples employed in study. (XLSX)

S2 Table Primers employed for AFX RABV amplification. (XLSX)

S3 Table Summary of the 161 sites subject to episodic selection as identified by MEME. (XLSX)

S4 Table Summary of predicted amino acid differences for the 133 AFX RABV database. (XLSX)
